# Inhibition of CD38 by 78c Enhanced NAD^+^, Alleviated Inflammation, and Decreased Oxidative Stress in Old Murine Macrophages Induced by Oral Pathogens

**DOI:** 10.3390/ijms26136180

**Published:** 2025-06-26

**Authors:** Kimberly Cao, Nityananda Chowdhury, Bridgette Wellslager, William D. Hill, Özlem Yilmaz, Hong Yu

**Affiliations:** 1 Department of Biomedical and Community Health Sciences, College of Dental Medicine, Medical University of South Carolina, Charleston, SC 29425, USA; caok@musc.edu (K.C.); chowdhun@musc.edu (N.C.); bridgette.wellslager@nih.gov (B.W.); yilmaz@musc.edu (Ö.Y.); 2 Department of Pathology and Laboratory Medicine, Medical University of South Carolina, Charleston, SC 29425, USA; hillwi@musc.edu

**Keywords:** CD38, NAD^+^, aging, cytokine, oxidative stress, periodontitis

## Abstract

CD38, a nicotinamide adenine dinucleotide (NAD^+^) glycohydrolase, increases in old murine macrophages after infection compared to young controls. We aimed to determine whether the increase in CD38 in old murine macrophages after infection is directly associated with enhanced inflammation induced by the oral pathogens *Aggregatibacter actinomycetemcomitans* (*Aa*) or *Porphyromonas gingivalis* (*Pg*) when compared to young controls. Additionally, we determined the effects of a specific CD38 inhibitor (78c) on CD38, NAD^+^, interleukin (IL)-1β, IL-6, tumor necrosis factor (TNF)-α expressions, and anti-oxidative responses in old murine macrophages induced by oral pathogens. Old and young murine macrophages were either uninfected or infected with the oral pathogens *Aa* or *Pg* for 1 to 24 h. Protein levels of CD38 and protein kinases, including nuclear factor kappa-B (NF-κB), phosphoinositide 3-kinase (PI3K), and mitogen-activated protein kinases (MAPKs), NAD^+^, and inflammatory cytokine (IL-1β, IL-6, TNF-α) levels were evaluated. Additionally, old murine macrophages were treated with a vehicle or a CD38 inhibitor (78c) and cells were either uninfected or infected with *Aa* or *Pg*. CD38, NAD^+^, cytokine (IL-1β, IL-6, TNF-α) levels, reactive oxygen species (ROS), NAPDH oxidase 1 (Nox1), and anti-oxidative enzymes, including superoxide dismutase1 (Sod1), glutathione peroxidase 4 (Gpx4), Peroxiredoxin 1 (Prdx1), thioredoxin reductase 1 (Txnrd1), and catalase (Cat), were evaluated. The results showed that old murine macrophages significantly enhanced CD38 and reduced NAD^+^ levels 24 h after *Aa* or *Pg* infection compared to young controls. This enhanced CD38 in old murine macrophages was not directly correlated with the activation of protein kinases (NF-κB, PI3K, and MAPKs), nor the (IL-1β, IL-6, TNF-α) levels in macrophages. The inhibition of CD38 by 78c reduced CD38, enhanced NAD^+^ levels, attenuated IL-1β, IL-6 and TNF-α pro-inflammatory cytokine levels, reduced ROS and Nox1 expressions, and enhanced expressions of Sod1, Gpx4, Prdx1, Txnrd1, and Cat in old murine macrophages infected with *Aa* or *Pg*. These results suggest that the inhibition of CD38 by 78c is a promising therapeutic strategy to treat aging-associated periodontitis.

## 1. Introduction

Cluster of Differentiation 38 (CD38) is a type 2 transmembrane glycoprotein ubiquitously expressed in most tissues and cells in mice and humans [1]. Predominantly, CD38 is expressed in immune cells, including B cells, T cells, plasma cells, natural killer cells, dendritic cells, monocytes, macrophages, and neutrophils [1]. CD38 is a nicotinamide adenine dinucleotide (NAD^+^) glycohydrolase, which breaks down NAD^+^ and generates nicotinamide (NAM) and ADP-ribose (ADPR). CD38 also degrades NAD^+^ via its ADP-ribosyl cyclase activity, which releases cyclic ADP-ribose (cADPR) [1,2,3,4,5]. NAD^+^ is expressed on the plasma membrane as well as on the intracellular organelle membranes, where NAD^+^ plays an important role in activating NAD^+^-dependent signaling pathways in different subcellular compartments [2,6]. NAD^+^ can be reduced to NADH via dehydrogenases, and NAD^+^ can be phosphorylated to NADP^+^ via NAD^+^ kinases [7]. The NAD^+^/NADH couple regulates cellular energy metabolism, glycolysis, and mitochondrial oxidative phosphorylation. In contrast, NADP^+^/NADPH maintains redox homeostasis and supports the biosynthesis of fatty and nucleic acids [7]. Additionally, NAD^+^ serves as a substrate for other NAD^+^ consuming enzymes, including sirtuins and poly-(ADP-Ribose) polymerases (PARPs) [8]. Sirtuins are NAD^+^-dependent histone deacetylases, which regulate diverse cellular processes including cellular metabolism, mitochondrial homeostasis, autophagy, DNA repair, apoptosis, oxidative stress, and inflammatory response [9,10]. In contrast, PARPs catalyze the covalent attachment of monomers or polymers of ADP-ribose units on a variety of amino acid residues on target proteins. PARPs play roles in DNA damage detection and repair, genomic stability, programmed cell death, and inflammation [11,12].

Individuals over 60 years of age accounted for 11% of the world population in 2016 and it is projected to reach 22% by 2050 [13]. NAD^+^ metabolism plays an essential role in senescence regulation and aging [2]. In older people, a decline occurs in the NAD^+^ level [2,14,15,16]. In contrast, an enhancement occurs in the CD38 levels during aging [17,18], which may be associated with increased aging-related inflammation through a process called inflammaging [19,20]. Increased CD38 during aging leads to further NAD^+^ depletion. Notably, decreased NAD^+^ in the aging population affects many aging-associated immune dysfunctions, including mitochondrial dysfunction, intracellular accumulation of oxidative damaged macromolecules (DNA, lipids, and proteins), dysregulated energy metabolism, impaired cellular “waste disposal”, impaired adaptive stress response, compromised DNA repair, dysregulated neuronal Ca^2+^ handling, stem cell exhaustion, and inflammation [14]. Therefore, the decline of NAD^+^ contributes to the pathogenesis of various aging-associated diseases, including infection, neurodegenerative diseases [14,15,16], cancer [3], and type 2 diabetes [3,15,21]. Hence, CD38 has become a therapeutic target for treating these aging-associated diseases [1,3,4,5,14,15,22].

Aging is associated with the development of many diseases, including periodontal disease, which is associated with comorbid systemic diseases, poor physical functioning, inflammatory dysregulation, and limited ability to self-care in frail older populations [23]. A previous study [24] showed that old (24-month-old) mice displayed significantly increased periodontal bone loss, accompanied by elevated expression of pro-inflammatory cytokines (IL-1β, IL-6, TNF-α, and IL-17) in the gingiva compared to young controls.

Oral bacterial pathogens, including *Aggregatibacter actinomycetemcomitans* (*Aa*, a major oral pathogen associated with 90% of localized aggressive periodontitis and 30% to 50% of severe adult periodontitis [25]) and *Porphyromonas gingivalis* (*Pg*, another major oral pathogen in the initiation and development of severe forms of chronic periodontal disease [26,27]) activate toll-like receptors (TLRs) and their downstream signaling pathways [28,29], including NF-κB, PI3K, and MAPKs [including extracellular signal-regulated kinases (ERKs), c-Jun N-terminal kinase (JNK), and p38 MAPK], leading to the production of pro-inflammatory cytokines [including IL-1β, IL-6, ΤNF-α, and receptor activator of NF-κB ligand (RANKL)]. These pro-inflammatory mediators subsequently cause periodontal tissue damage and alveolar bone loss.

Additionally, oral bacterial pathogens can modulate the generation of reactive oxygen species (ROS) in specific cell types by activating NADPH oxidases (NOXs), which play roles in host defense to eliminate infected bacterial pathogens [26,27]. However, excessive ROS causes oxidative stress, contributing to mitochondrial dysfunction in aging individuals [30]. The innate immune response also possesses many anti-oxidative enzymes [including superoxide dismutase1 (Sod1), glutathione peroxidase 4 (Gpx4), Peroxiredoxin 1 (Prdx1), thioredoxin reductase 1 (Txnrd1), and catalase (Cat)], which reduce oxidative stress induced by bacterial pathogens [7]. NADP^+^/NADPH serve as coenzymes in the anti-oxidative response to maintain cellular redox homeostasis [7,31]. NAD^+^ depletion with aging caused mitochondrial dysfunction, a decline in energy production, and accumulation of ROS that produce high oxidative stress [6].

A previous study [17] showed that old (18-month-old) murine bone marrow-derived monocytes and macrophages (BMMs) displayed higher CD38 protein levels when stimulated by various doses (0.5 to 50 ng/mL) of bacterial lipopolysaccharide (LPS) for 20 h compared to young (3-month-old) mice controls. However, it was not clear if the enhanced CD38 levels in old murine BMMs were directly correlated to enhanced pro-inflammatory cytokine levels in old murine BMMs. Additionally, it was not clear if the inhibition of CD38 by a CD38-specific inhibitor (78c) in old murine BMMs could attenuate pro-inflammatory cytokine levels, enhance NAD^+^ expression, and reduce the oxidative stress induced by oral pathogens.

In the present study, we first determined if there were differences in CD38 and NAD^+^ levels between young and old murine BMMs with or without infection with the oral pathogens *Aa* or *Pg*. Next, we determined if CD38 protein expression was directly correlated with the activated NF-κB, PI3K, and MAPKs protein levels or the enhanced pro-inflammatory cytokine (IL-1β, IL-6, ΤNF-α) levels in old murine BMMs when compared to young controls. Finally, we evaluated the effects of a CD38-specific inhibitor (78c) in CD38 and NAD^+^ levels, pro-inflammatory cytokine expression, and oxidative stress in old murine BMMs induced by oral pathogens.

## 2. Results

### 2.1. Old Murine BMMs Exhibited Significantly Higher CD38 Protein and Lower NAD^+^ Expressions After Infection with Oral Pathogens Aa or Pg Compared with Young Controls

**Figure 1:**
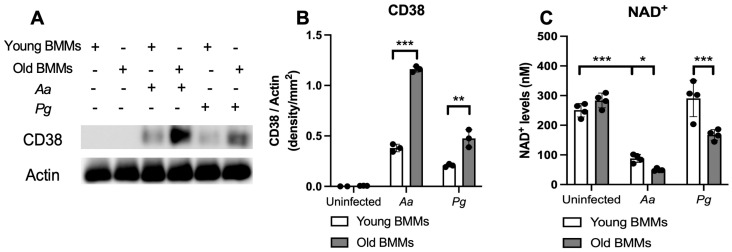
Old murine BMMs exhibited significantly higher CD38 protein and lower NAD^+^ expressions after infection with either *Aggregatibacter actinomycetemcomitans* (*Aa*) or *Porphyromonas gingivalis* (*Pg*) than young controls. Young and old murine BMMs were either uninfected or infected with an oral bacterial pathogen *Aa* or *Pg* (MOI 20) for 24 h. (**A**) Protein levels of CD38 and pan-actin in cell lysate were determined by Western Blot. (**B**) Protein densitometry of CD38 were quantified compared with control actin expression (*n* = 3). (**C**) NAD^+^ levels in murine BMMs with or without bacterial infection (*n* = 4). Statistics were analyzed by ordinary one-way ANOVA with Tukey’s multiple comparisons test. (* *p* < 0.05, ** *p* < 0.01, *** *p* < 0.001).

First, we compared CD38 protein levels in young vs. old murine BMMs with or without infection with the oral pathogens *Aa* or *Pg*. As shown in Figure 1A,B, CD38 protein levels were undetectable in uninfected young and old murine BMMs. In contrast, 24 h after *Aa* or *Pg* infection, old murine BMMs exhibited significantly higher CD38 protein levels compared with young controls (** *p* < 0.01, *** *p* < 0.001). Compared with young controls, old murine BMMs displayed an average of 3.1-fold and 2.3-fold of CD38 protein levels after *Aa* or *Pg* infection, respectively. Accordingly, in uninfected young or old murine BMMs, the NAD^+^ levels were similar between young and old murine BMMs. In contrast, in old murine BMMs infected with *Aa* or *Pg* for 24 h, the NAD^+^ levels were significantly lower in old murine BMMs than young controls (* *p* < 0.05, *** *p* < 0.001). Compared with young controls, the NAD^+^ levels were reduced by about 43.6% and 42.3% in old murine BMMs infected with *Aa* or *Pg*, respectively. These results support that old murine BMMs expressed abnormally higher CD38 protein levels and lower NAD^+^ levels after infection with the oral pathogens *Aa* or *Pg* compared with young controls.

### 2.2. The Abnormal High CD38 Protein Level in Old Murine BMMs After Infection with the Oral Pathogens Aa or Pg Was Not Directly Correlated with the Level of Immune Responses in Old Murine BMMs Compared with Young Controls

**Figure 2:**
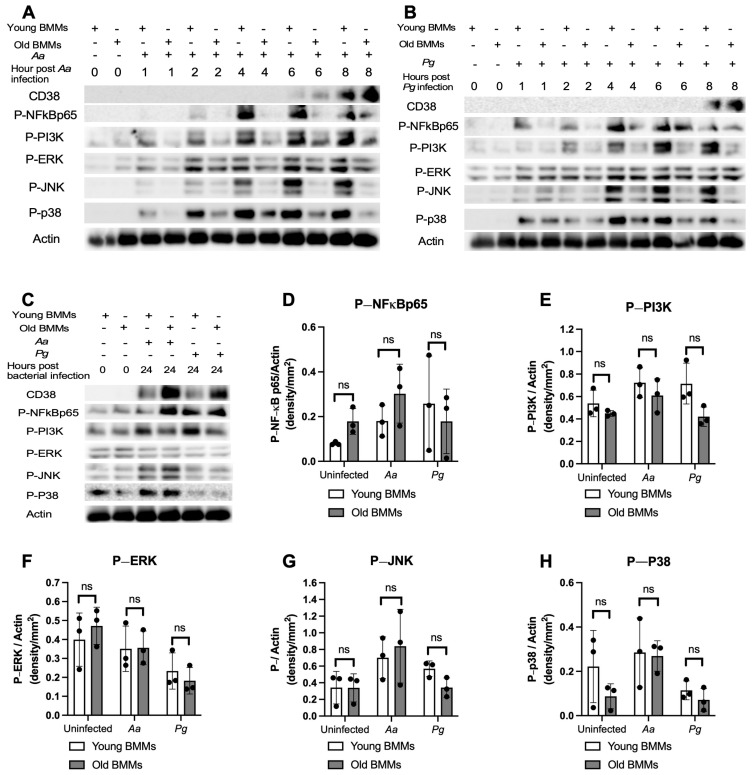
Old murine BMMs displayed delayed immune responses to infection with either the oral pathogens *Aggregatibacter actinomycetemcomitans* (*Aa*) or *Porphyromonas gingivalis* (*Pg*) compared to young controls. Young and old murine BMMs were either uninfected or infected with an oral bacterial pathogen *Aa* or *Pg* for 1 to 24 h. (**A**–**C**) Protein levels of CD38, p-NFκBp65, p-PI3K, p-ERK, p-JNK, p-p38, and pan-actin in cell lysate were determined by Western Blot. Protein densitometry of p-NFκBp65 (**D**), p-PI3K (**E**), p-ERK (**F**), p-JNK (**G**), and p-p38 (**H**) 24 h after bacterial infection were evaluated. Statistics were analyzed by ordinary one-way ANOVA with Tukey’s multiple comparisons test. (*n* = 3, ns: no significance).

**Figure 3:**
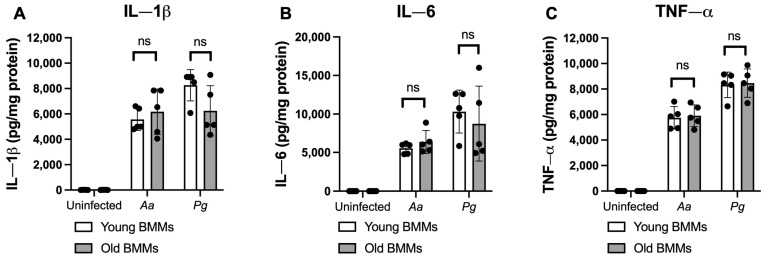
Old murine BMMs displayed similar IL-1β, IL-6, and TNF-α cytokine levels 24 h after infection with either the oral pathogens *Aggregatibacter actinomycetemcomitans* (*Aa*) or *Porphyromonas gingivalis* (*Pg*) compared to young controls. Young and old murine BMMs were either uninfected or infected with an oral bacterial pathogen *Aa* or *Pg* (MOI 20) for 24 h. Cytokine levels of IL-1β (**A**), IL-6 (**B**), and TNF-α (**C**) were quantified by ELISA and calibrated by protein expression in cell lysate. Statistics were analyzed by ordinary one-way ANOVA with Tukey’s multiple comparisons test. (*n* = 5, ns: no significance).

To determine if old murine BMMs displayed an abnormally high immune response to oral pathogenic infection that contributes to the high CD38 expression in old murine BMMs after infection with an oral pathogen, we quantified the protein levels of CD38 and some protein kinases (including NF-κB, PI3K, and MAPKs) by Western blot in old and young murine BMMs at various time points (1, 2, 4, 6, 8, and 24 h) after infection with *Aa* or *Pg*. As shown in Figure 2A,B, the old murine BMMs displayed a delayed activation in p-NF-κBp65, p-PI3K, p-ERK, p-JNK, and p-p38 MAPK compared with the young controls. The activation of NF-κB, PI3K, and MAPKs occurred at an early time point (1 h) in young murine BMMs after *Aa* or *Pg* infection. In contrast, old murine BMMs displayed weak activations of p-NF-κBp65, p-PI3K, p-ERK, p-JNK, and p-p38 MAPK at all time points (1 h to 8 h) except 24 h after *Aa* or *Pg* infection. Although old murine BMMs displayed a delayed immune response to *Aa* or *Pg* infection, the old murine BMMs still expressed higher detectable CD38 protein levels at 8 h after bacterial infection compared with the young controls (Figure 2A,B). Although the old murine BMMs exhibited higher CD38 protein expression at 24 h than the young controls, the old murine BMMs showed similar levels of p-NF-κBp65, p-PI3K, p-ERK, p-JNK, and p-p38 at 24 h following *Aa* or *Pg* infection compared with the young controls (Figure 2C–H, ns: no significance). Additionally, quantification of the levels of IL-1β, IL-6, and TNF-α pro-inflammatory cytokines (Figure 3) showed that old murine BMMs displayed similar levels of IL-1β, IL-6, and TNF-α 24 h after infection with *Aa* or *Pg* compared with controls in young BMMs (Figure 3A–C). These results support the conclusion that the abnormally high CD38 expression in old murine BMMs was not directly correlated with the activation of NF-κB, PI3K, and MAPK protein kinases, nor the IL-1β, IL-6, and TNF-α pro-inflammatory cytokine levels in old murine BMMs.

### 2.3. Inhibition of CD38 by 78c Suppressed CD38, NF-κB, PI3K, and MAPK Protein Kinases, Enhanced NAD^+^, and Attenuated IL-1β, IL-6, and TNF-α Pro-Inflammatory Cytokine Levels in Old Murine BMMs Infected with Oral Pathogens Aa or Pg

**Figure 4:**
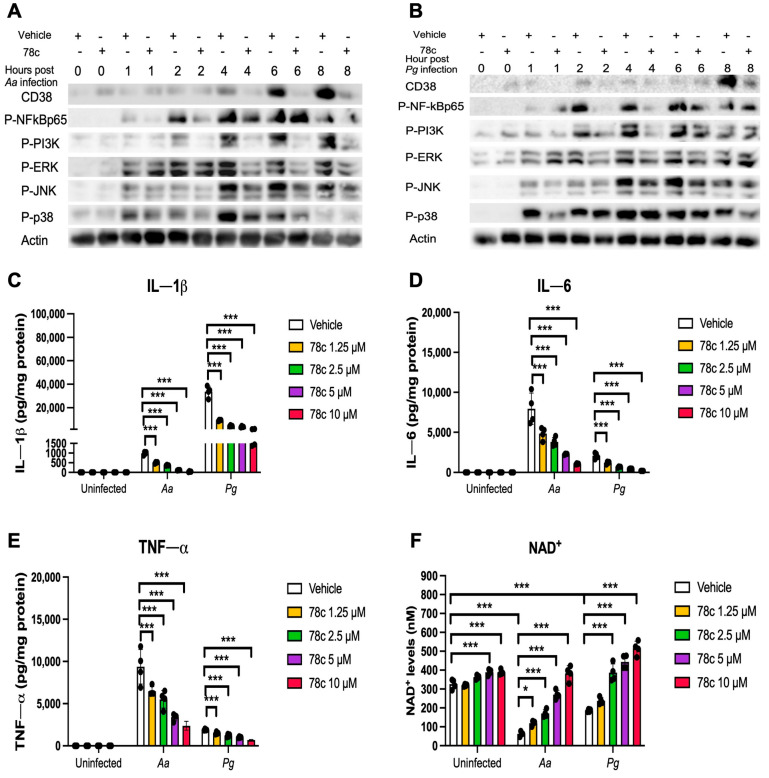
Inhibition of CD38 by 78c suppressed CD38, NF-κB, PI3K, and MAPKs; attenuated IL-1β, IL-6, and TNF-α pro-inflammatory cytokine levels; and enhanced NAD^+^ in old murine BMMs after infection with either the oral pathogens *Aggregatibacter actinomycetemcomitans* (*Aa*) or *Porphyromonas gingivalis* (*Pg*). Old murine BMMs were treated with vehicle or 78c (10 μM) with or without infection with the oral bacterial pathogen *Aa* or *Pg* for 1 to 8 h. Protein levels of CD38, p-NFκBp65, p-PI3K, p-ERK, p-JNK, p-p38, and pan-actin in cell lysate induced by *Aa* (**A**) or *Pg* (**B**) were determined by Western Blot. Old murine BMMs were treated with vehicle (diluted DMSO) or 78c (1.25 to 10 μM) with or without infection of *Aa* or *Pg* for 24 h. Cytokine levels of IL-1β (**C**), IL-6 (**D**), and TNF-α (**E**) were quantified by ELISA and calibrated by protein expression in cell lysate. (**F**) NAD^+^ levels were measured and calibrated by cell growth and viability. Statistics were analyzed by one-way ANOVA with Dunnett’s multiple comparisons test (*n* = 4, * *p* < 0.05, *** *p* < 0.001).

Previously, our study [32] demonstrated that the inhibition of CD38 by 78c reduced CD38 and attenuated the activation of NF-κB, PI3K, and MAPKs induced by the oral pathogens *Aa* or *Pg* in murine BMMs derived from TALLYHO/JngJ mice (type 2 diabetic mice). We hypothesized that treatment with 78c would also suppress CD38 and the activation of NF-κB, PI3K, and MAPKs induced by the oral pathogens *Aa* or *Pg* in old murine BMMs. As shown in Figure 4A,B, treatment with 78c (10 μM) reduced CD38, p-NF-κBp65, p-PI3K, p-ERK, p-JNK, and p-p38 MAPK induced by *Aa* or *Pg*. Accordingly, we observed reductions in IL-1β, IL-6, and TNF-α pro-inflammatory cytokines in cells treated with 78c compared with the controls. Treatment with 78c (1.25 μM, 2.5 μM, 5 μM, and 10 μM) in old murine BMMs reduced IL-1β by 50.1%, 63.9%, 88.7%, and 96.5%, respectively, as induced by *Aa*, and attenuated IL-1β by 73.7%, 87.3%, 89.6%, and 95.6%, respectively, as induced by *Pg* (Figure 4C). Treatment with 78c (1.25 μM, 2.5 μM, 5 μM, and 10 μM) also reduced IL-6 by 40.6%, 53.2%, 70.9%, and 88.1%, respectively, as induced by *Aa*, and attenuated IL-6 by 35.2%, 68.4%, 82.2%, and 90.4%, respectively, as induced by *Pg* (Figure 4D). Additionally, treatment with 78c (1.25 μM, 2.5 μM, 5 μM, and 10 μM) reduced TNF-α by 36.7%, 44.3%, 65.7%, and 75.4%, respectively, as induced by *Aa*, and attenuated TNF-α by 21.4%, 36.8%, 47.1%, and 64.1%, respectively, as induced by *Pg* (Figure 4E). In uninfected old murine BMMs, treatment with 78c (5 μM and 10 μM) enhanced NAD^+^ by 1.2-fold and 1.3-fold, respectively (Figure 4F). The NAD^+^ levels declined about 82.8% in cells treated with vehicle and infected with *Aa* and decreased about 42.9% in cells treated with vehicle and infected with *Pg* as compared with the NAD^+^ levels in cells treated with vehicle without bacterial infection (Figure 4F). Treatment with 78c (1.25 μM, 2.5 μM, 5 μM, and 10 μM) enhanced NAD^+^ by 1.9-fold, 2.7-fold, 4.3-fold, and 6.6-fold, respectively, in old murine BMMs infected with *Aa* and enhanced NAD^+^ by 1.3-fold, 2.1-fold, 2.4-fold, and 3.0-fold, respectively, in old murine BMMs infected with *Pg* (Figure 4F). These data support that the inhibition of CD38 by 78c suppressed CD38 and prevented the decline of NAD^+^ induced by oral pathogens. Additionally, the inhibition of CD38 by 78c suppressed the activation of NF-κB, PI3K, and MAPKs as induced by the oral pathogens *Aa* or *Pg,* and subsequently attenuated IL-1β, IL-6, and TNF-α pro-inflammatory cytokines as induced by oral pathogens.

### 2.4. Inhibition of CD38 by 78c Reduced Oxidative Stress in Old Murine BMMs Infected with Oral Pathogens

**Figure 5:**
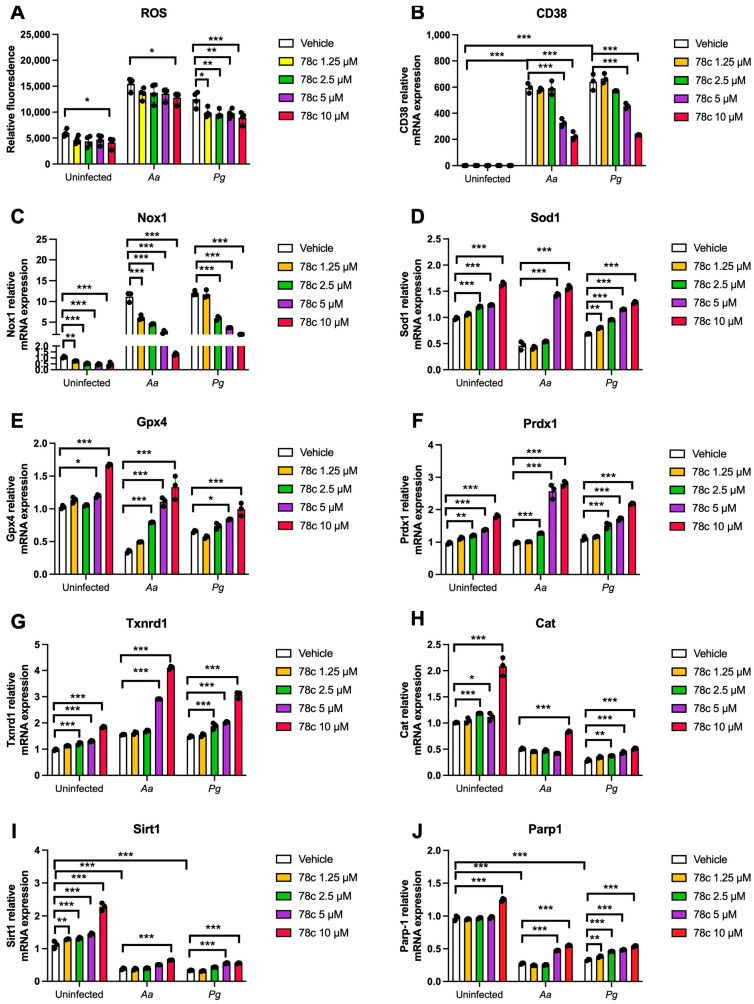
Inhibition of CD38 by 78c reduced reactive oxygen species (ROS) and Nox1, but enhanced anti-oxidant enzymes (Sod1, Gpx4, Prdx1, Txnrd1, and Cat), Sirt1, and Parp1 mRNA levels in old murine BMMs either uninfected or infected with either the oral pathogens *Aggregatibacter actinomycetemcomitans* (*Aa*) or *Porphyromonas gingivalis* (*Pg*). Old murine BMMs were treated with vehicle or 78c (1.25 to 10 μM) with or without infection with either the oral bacterial pathogen *Aa* or *Pg* for 24 h (ROS study) or 8 h (RT-qPCR study). (**A**) ROS was detected by measuring fluorescence in cells using a CellROX^TM^ Green reagent and calibrated by cell growth and viability (*n* = 4). (**B**) CD38 mRNA, (**C**) Nox1 mRNA, (**D**) Sod1 mRNA, (**E**) Gpx4 mRNA, (**F**) Prdx1 mRNA, (**G**) Txnrd1 mRNA, (**H**) Cat mRNA, (**I**) Sirt1 mRNA, and (**J**) Parp1 mRNA levels were quantified using RT-PCR and normalized by β-actin expression (*n* = 3). Statistics were analyzed using an ordinary one-way ANOVA with Dunnett’s multiple comparisons test (* *p* < 0.05, ** *p* < 0.01, *** *p* < 0.001).

**Figure 6:**
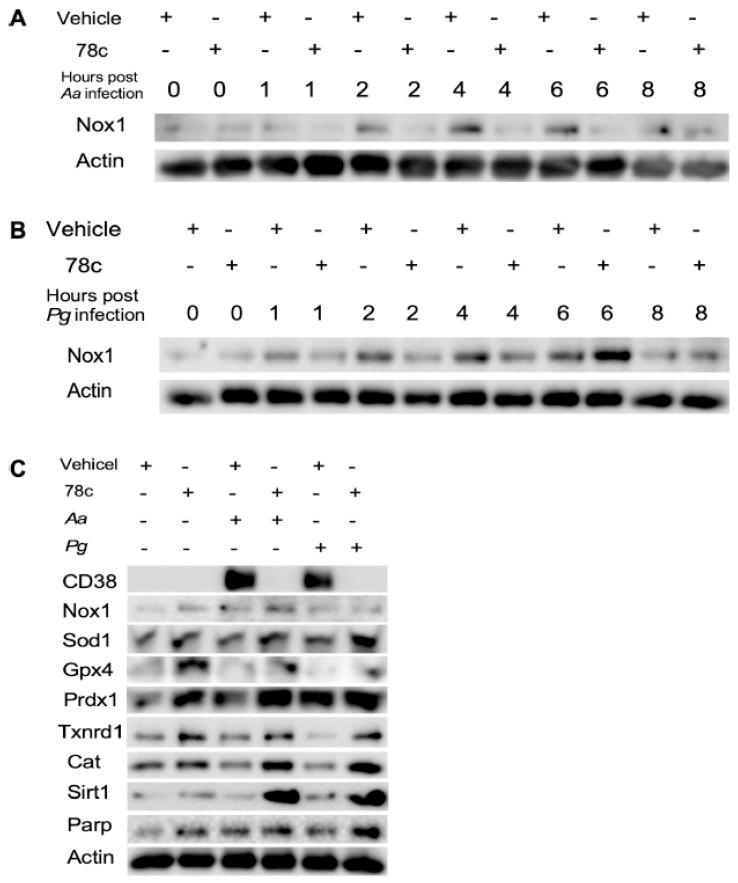
Inhibition of CD38 by 78c inhibited Nox1, but enhanced anti-oxidant enzymes (Sod1, Gpx4, Prdx1, Txnrd1, and Cat), Sirt1, and Parp1 protein levels in old murine BMMs either uninfected or infected with the oral pathogens *Aggregatibacter actinomycetemcomitans* (*Aa*) or *Porphyromonas gingivalis* (*Pg*). Old murine BMMs were treated with vehicle or 78c (10 μM) with or without infection of either of the oral bacterial pathogens *Aa* or *Pg* for various time points (1, 2, 4, 6, 8, or 24 h). Nox1 and pan-actin protein expressions in old murine BMMs infected with *Aa* (**A**) or *Pg* (**B**) were evaluated by Western blot. CD38, Nox1, Sod1, Gpx4, Prdx1, Txnrd1, Cat, Sirt1, Parp, and pan-actin protein expression (**C**) in old murine BMMs either uninfected or infected with *Aa* or *Pg* for 24 h were evaluated by Western blot.

Since excessive ROS causes oxidative stress, contributing to mitochondrial dysfunction in aging [30], and NADP^+^/NADPH serve as coenzymes in the anti-oxidative response to maintain cellular redox homeostasis [7,31], we hypothesized that the inhibition of CD38 by 78c in old murine BMMs could increase NAD^+^ and subsequently reduce oxidative stress induced by oral pathogens. As shown in Figure 5A, treatment with 78c (10 μM) for 24 h significantly reduced ROS in old murine BMMs either with or without infection of *Aa* (* *p* < 0.05) compared with vehicle controls. Treatment with 78c (1.25 to 10 μM) for 24 h also significantly reduced ROS in old murine BMMs infected with *Pg* (* *p* < 0.05, ** *p* < 0.01, *** *p* < 0.01). To determine how 78c regulates oxidative response and affects aging-associated immune responses in old murine BMMs, we quantified the mRNA levels of CD38, Nox1, anti-oxidative enzymes (Sod1, Gpx4, Prdx1, Txnrd1, and Cat), and NAD^+^ consuming enzymes (Sirt1 and Parp1) in old murine BMMs that were either left uninfected or were infected with *Aa* or *Pg* for 8 h. Treatment with 78c (5 and 10 μM) reduced CD38 mRNA levels by 44.5% and 61.2% as induced by *Aa*, respectively, and reduced CD38 mRNA levels by 28.5% and 63.2% as induced by *Pg*, respectively (Figure 5B). Treatment with 78c dose-dependently reduced Nox1 as induced by *Aa* or *Pg* compared with the controls (Figure 5C). Additionally, treatment with 78c dose-dependently enhanced anti-oxidant enzyme (Sod1, Gpx4, Prdx1, Txnrd1, and Cat) mRNA levels in old murine BMMs (Figure 5D–H). Furthermore, treatment with 78c does-dependently enhanced two NAD^+^ consuming enzyme (Sirt1 and Parp1) mRNA levels in old murine BMMs either uninfected or infected with *Aa* or *Pg* compared with controls (Figure 5I,J). Western blot protein assay also showed that treatment with 78c (10 μM) in old murine BMMs suppressed Nox1 protein levels at 1, 2, 4, and 6 h after *Aa* infection compared with the controls (Figure 6A), and reduced Nox1 protein expressions at 1, 2, and 4 h after *Pg* infection compared with the controls (Figure 6B). After treatment with 78 c (10 μM) for 24 h, CD38 protein levels were reduced in old murine BMMs infected with *Aa* or *Pg* compared with the controls (Figure 6C). At 24 h after treatment, Nox1 protein levels were similar between 78c-treated cells and vehicle-treated cells. We observed enhanced protein levels of anti-oxidant enzymes (Sod1, Gpx4, Prdx1, Txnrd1, and Cat) in uninfected old murine BMMs or *Aa*- or *Pg*-infected BMMs compared with the controls (Figure 6C). These results support that 78c attenuated oxidative stress by inhibiting Nox1 mRNA and protein expressions, while enhancing anti-oxidant enzyme mRNA and protein expressions. Additionally, we also observed enhanced NAD^+^ consuming enzymes (Sirt1 and Parp) proteins in 78c (10 μM)-treated uninfected old murine BMMs or *Aa*- or *Pg*-infected BMMs compared with the controls (Figure 6C).

### 2.5. Knockdown of CD38 by CD38 shRNA Increased NAD^+^ and Reduced Oxidative Stress in Old Murine BMMs Infected with Oral Pathogens

**Figure 7:**
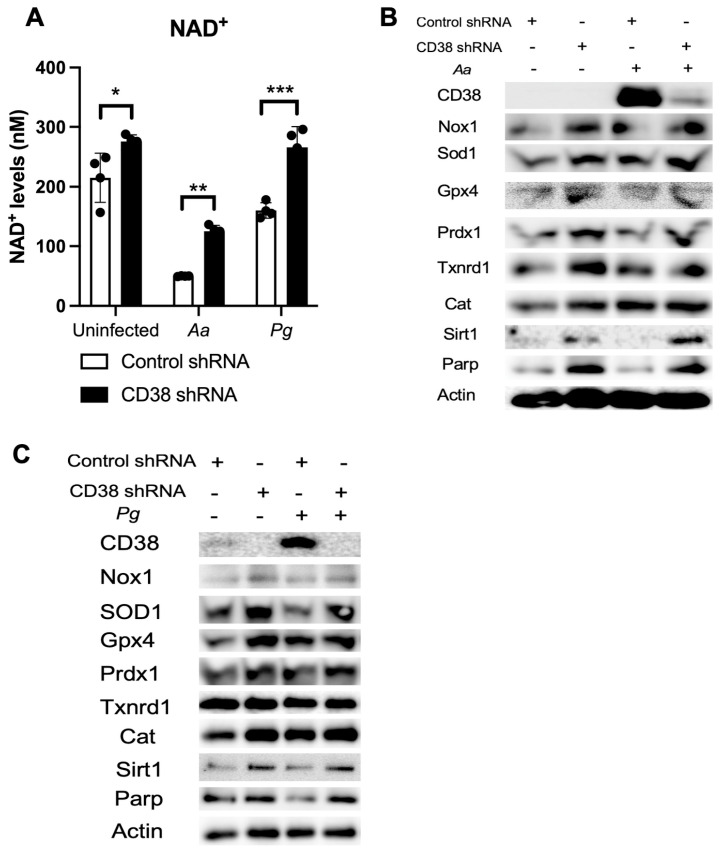
Knockdown of CD38 by a CD38 shRNA increased NAD^+^ and enhanced the protein levels of Nox1, Sod1, Gpx4, Prdx1, Txnrd1, Cat, Sirt1, and Parp1 in old murine BMMs either uninfected or infected with the oral pathogens *Aggregatibacter actinomycetemcomitans* (*Aa*) or *Porphyromonas gingivalis* (*Pg*). Old murine BMMs were treated with a CD38 shRNA or a control shRNA for 72 h. Then, the cells were either uninfected or infected with the oral pathogens *Aa* or *Pg* for 24 h for the NAD^+^ assay (**A**) or for 6 h for Western blot protein assay (**B**,**C**). CD38, Nox1, Sod1, Gpx4, Prdx1, Txnrd1, Cat, Sirt1, Parp, and control pan-actin protein expression were evaluated by Western blot. (* *p* < 0.05, ** *p* < 0.01, *** *p* < 0.001).

Our previous study [32] showed that treatment with a CD38 shRNA reduced CD38 expression and significantly increased NAD^+^ levels in murine BMMs derived from TALLYHO/JngJ mice when the cells were either uninfected, infected with the oral pathogens (*Aa* or *Pg*), or stimulated by advanced end-products (AGEs). Similarly, treatment with the CD38 shRNA also significantly enhanced NAD^+^ levels in old murine BMMs when the cells were either uninfected or infected with the oral pathogens compared to the controls (Figure 7A). Additionally, treatment with the CD38 shRNA slightly increased the protein levels of Nox1, Sod1, Gpx4, Prdx1, Txnrd1, Cat, Sirt1, and Parp in old murine BMMs when the cells were uninfected or infected with the oral pathogens *Aa* or *Pg* (Figure 7B,C). These studies demonstrated the role of NAD^+^ in enhancing anti-oxidative responses and promoting longevity gene (Sirt1 and Parp) expressions.

## 3. Discussion

Periodontitis is an inflammatory bone loss disease. Oral bacterial pathogens not only induce the generation of IL-1β, IL-6, and TNF-α, but also RANKL, the major osteoclast differentiation factor [33]. RANKL binds with its receptor RANK, which promotes osteoclast precursors (monocytes and macrophages) to differentiate and fuse to form multinucleated osteoclasts, leading to alveolar bone loss. In the current study, we demonstrated that old murine BMMs exhibited an abnormal immune response to infection with the oral pathogens *Aa* or *Pg*, including a delayed activation of NF-κB, PI3K, and MAPK protein kinases, an enhanced CD38 expression, and a reduced NAD^+^ expression compared with the controls in young murine BMMs (Figure 1 and Figure 2A,B). Our study is in accordance with a previous study [17], which showed that old murine BMMs displayed higher CD38 protein expressions when stimulated by LPS for 20 h compared with young mice controls. Previously, Chini et al. [34] showed that inducing senescence in human umbilical vain endothelial cells (HUVECs) by DNA damage through exposure to X-ray irradiation or gamma irradiation enhanced markers for senescence, including p21, p16^Ink4a^, and PAI1. However, CD38 mRNA was not induced by these treatments [34]. Instead, Chini et al. [34] discovered that senescent cells induced by either X-ray irradiation or gamma irradiation secreted higher inflammatory cytokines, including IL6, IL-8, and monocyte chemoattractant protein -1 (MCP1) than the controls. When murine BMMs were incubated with conditioned media derived from senescent cells induced by either X-ray irradiation or gamma irradiation, the mRNA and protein levels of CD38 were induced [34]. In the current study, the high CD38 expression in old murine BMMs after infection with oral pathogens was not directly correlated with the activation of NF-κB, PI3K, and MAPK protein kinases nor the amount of pro-inflammatory cytokine (IL-1β, IL-6, and TNF-α) released in old murine BMMs.

Our previous study [32] demonstrated that treatment with 78c suppressed NF-κB, PI3K, and MAPK protein kinases induced by the oral pathogens *Aa* or *Pg* in murine BMMs derived from TALLYHO/JngJ mice (type 2 diabetic mice). In accordance with our previous study, treatment with 78c also reduced the activation of NF-κB, PI3K, and MAPK protein kinases and attenuated the levels of the pro-inflammatory cytokine (IL-1β, IL-6, TNF-α) that were induced by the oral pathogens *Aa* or *Pg* in old murine BMMs (Figure 4A–E). In contrast, our previous study [32] showed that treatment with a CD38 shRNA significantly reduced CD38 and increased NAD^+^ levels after murine BMMs infected with oral pathogens or stimulated by AGEs. Treatment with the CD38 shRNA only slightly reduced IL-1β, IL-6, and TNF-α levels induced by AGEs. Treatment with the CD38 shRNA displayed limited effects in suppressing IL-1β, IL-6, and TNF-α levels induced by the oral pathogens *Aa* or *Pg* compared to the controls. These results suggested that the CD38 inhibitor (78c) might possess some off-target effects in suppressing pro-inflammatory cytokine levels induced by the oral pathogens compared to the CD38 shRNA treatment.

Because NADPH oxidase (Nox) activation is associated with sensing the molecular signatures of microbial pathogens by TLRs [35] and ROS-mediated cellular signal pathways interplay with TLRs downstream signaling pathways (including NF-κB, PI3K, and MAPK protein kinases) [36], the inhibition of NF-κB, PI3K, and MAPK protein kinases by 78c could reduce Nox1 expression induced by oral pathogens (Figure 6A,B). The enhancement of anti-oxidant enzymes (including Sod1, Gpx4, Prdx1, Txnrd1, and Cat) in murine BMMs treated with 78c (Figure 5D–H and Figure 6C) was caused by the increase in NAD^+^ levels in 78c-treated cells (Figure 4F). As NADPH/NADP^+^ are involved in maintaining redox homeostasis by turning O_2_^−^ into H_2_O_2_ by Sod1 and subsequently turning H_2_O_2_ into H_2_O by other anti-oxidant enzymes (including Gpx4, Prdx1, Txnrd1, and Cat) [7,31], the inhibition of the degradation of NAD^+^ by CD38 in 78c-treated murine BMMs could enhance NAD^+^ and subsequently increase these anti-oxidant enzyme expressions. Our CD38 shRNA studies also confirmed that the knockdown of CD38 by the CD38 shRNA enhanced NAD^+^ levels and subsequently increased anti-oxidative enzyme protein levels (Figure 7). Our findings are in accordance with a prior study [37], which demonstrated that the inhibition CD38 by apigenin (a flavonoid with CD38 inhibitory activity) ameliorated oxidative stress by enhancing Sod and Gpx expressions in the skeletal muscles of aged mice.

Additionally, our previous study [32] demonstrated that treatment with 78c suppressed osteoclastogenesis and bone resorption induced by RANKL. Mechanistically, we demonstrated that treatment with 78c reduced podosome (basic cell adhesion unit) components (including PI3K, Pyk2, Src, F-actin, integrins, paxillin, and talin) induced by RANKL. Therefore, treatment with 78c could potentially alleviate inflammatory bone loss in patients with periodontitis. In contrast, our previous study [32] showed that treatment with the CD38 shRNA increased osteoclastogenesis and bone resorption induced by RANKL compared to the control shRNA treatment. These results suggested that the CD38 inhibitor (78c) possessed some off-target effects in suppressing osteoclastogenesis and bone resorption induced by RANKL compared to the CD38 shRNA treatment.

Previous studies [10,38] demonstrated that sirtuins play roles in extending the lifespan of organisms. Numerous studies reported that SIR2, the first identified sirtuin protein in yeast, extended the lifespan in yeast [39], C. elegans [40], and Drosophila [41]. Sirt1 is the most studied and the mammalian closest ortholog to SIR2. Sirt1 expression declines with aging in animals and human tissues [42]. In contrast, the over-expression of Sir1 in the brain extended the lifespan of mice [43]. In the current study, treatment with 78c enhanced NAD^+^ (Figure 4F) and subsequently increased Sirt1 mRNA and protein expressions in old murine BMMs either uninfected or infected with oral pathogens (Figure 5I and Figure 6C), supporting that treatment with 78c is a promising therapeutic approach to treat aging-associated periodontitis, which can enhance NAD^+^ and Sirt1, maintain mitochondrial homeostasis and metabolic function, and promote longevity. In response to the increase in NAD^+^ in 78c-treated cells, we also observed enhanced Parp1 mRNA (Figure 5J) and Parp protein levels (Figure 6C) in old murine BMMs treated with 78c. This enhanced Parp could assist in repairing damaged DNA in cells.

Previously, accumulated evidence suggests that NAD^+^ levels decline with aging at a systemic level in diverse organisms, including rodents and humans, contributing to the development of many aging-associated diseases [2,15,44,45]. These enhanced NAD^+^ levels in the aging population are associated with chronic inflammation in aging patients, called inflammaging [19,20]. In the current study, the mice were bred in a specific pathogen-free condition and were relatively healthy without inflammation. Therefore, we did not detect CD38 protein levels in uninfected murine BMMs, and uninfected old murine BMMs expressed similar levels of NAD^+^ compared with the young controls. Because human bodies are exposed to varieties of pathogens and aging patients often have comorbidity with various chronic inflammation (including atherosclerosis, cardiovascular events, cancer, autoimmune diseases), aging patients could have high levels of CD38^+^ and reduced levels of NAD^+^ compared with the young controls.

A previous study [24] showed that old mice (24 months old) and young mice (3 months old) displayed similar numbers of macrophages or osteoclasts in the periodontal tissues during the disease induction period. However, after depletion of the macrophages by administration of PLX3397 (an inhibitor of macrophage colony stimulating factor 1 receptor) in the recovery period, old mice resulted in decreased inflammatory cytokines within the gingiva and decreased bone loss, whereas macrophage depletion in young mice resulted in no beneficial or detrimental effects [24]. These results suggested that the enhanced expressions of pro-inflammatory cytokines and increased bone loss observed in old mice might be attributed to the defected immune functions in old macrophages in the disease recovery period. Wu et al. [46] also showed that old mice exhibited less bacterial diversity and enhanced *Pg* colonization compared to the young controls, which might also contribute to increased inflammation and alveolar bone loss in old mice.

**Figure 8:**
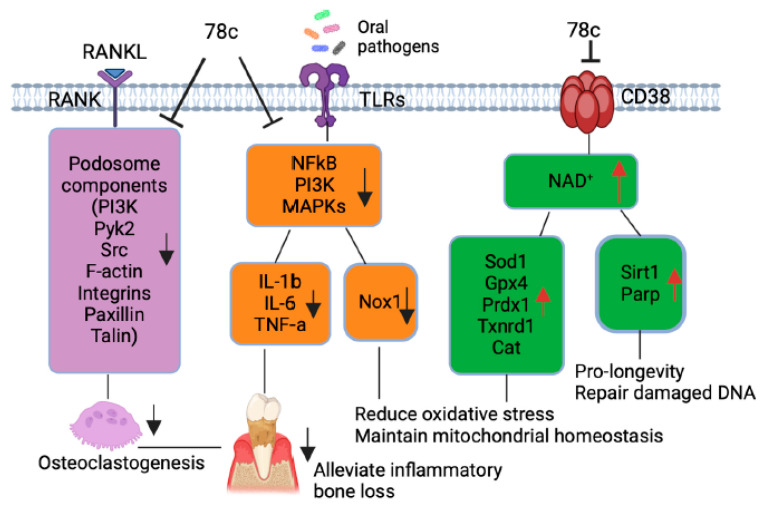
Roles of CD38 inhibitor (78c) in treating aging-associated with periodontitis. Treatment with 78c suppressed podosome component (PI3K, Pyk2, Src, F-actin, integrins, paxillin, and talin) expressions, subsequently inhibiting osteoclastogenesis and bone resorption [32]. Treatment with 78c inhibited NF-κB, PI3K, and MAPK protein kinases induced by oral bacterial pathogens, suppressing IL-1β, IL-6, TNF-α, and inflammation. Treatment with 78c reduced Nox1 expression, increased NAD^+^, and enhanced anti-oxidant enzyme (Sod1, Gpx4, Prdx1, Txnrd1, and Cat) expression and subsequently reduce oxidative stress and maintain mitochondrial homeostasis. Treatment with 78c increased NAD^+^ consuming enzyme (Sirt1 and Parp) expressions, which subsequently promote longevity and the repair of DNA damage.

Scaling and root surface debridement are the traditional “gold standard” treatment for stages I-III of periodontitis. There are still patients or sites that show poor response to non-surgical periodontal treatment and long-term supportive maintenance efforts. This could be due to sustained dysbiosis, bacteria invasion to periodontal tissues, or a non-resolving chronic inflammatory response. Previous studies [47,48] demonstrated that treatment with 78c in aged mice reversed age-related NAD^+^ decline and increased the lifespan and health span of naturally aged mice. Treatment with 78c improved several physiological and metabolic aging parameters, including glucose tolerance, muscle function, exercise capacity, and cardiac function in natural and accelerated aging mouse models [47,48]. Our previous study [32] demonstrated that the inhibition of CD38 by 78 attenuated IL-1β, IL-6, and TNF-α pro-inflammatory cytokine expressions induced by the oral pathogens *Aa* or *Pg* in murine BMMs derived from TALLYHO/JngJ mice (type 2 diabetic mice). Additionally, treatment with 78c reduced osteoclastogenesis and bone resorption induced by RANKL (Figure 8) [32]. In the current study, we also showed that treatment with 78c in old murine BMMs inhibited NF-κB, PI3K, and MAPK protein kinases as induced by the oral pathogens *Aa* or *Pg*, and subsequently alleviated IL-1β, IL-6 and TNF-α pro-inflammatory cytokine expressions and Nox1 mRNA and protein expressions. Additionally, treatment with 78c suppressed CD38 and enhanced NAD^+^ levels, and subsequently increased the mRNA and protein levels of anti-oxidant enzymes (Sod1, Gpx4, Prdx1, Txnrd1, and Cat) in old murine BMMs either uninfected or infected with the oral pathogens *Aa* or *Pg*. Our and other studies support that the inhibition of CD38 by 78c could serve as an adjunctive therapy for aging associated diseases, such as aging-associated periodontitis, to inhibit periodontal inflammation, attenuate osteoclastogenesis and alveolar bone resorption, alleviate oxidative stress, and prolong the health span of human beings.

The current study has some limitations. Although we showed that old murine BMMs displayed higher CD38 protein levels after infection with the oral pathogens *Aa* or *Pg*, it was not clear which mechanisms were associated with the increase in CD38 in old murine BMMs. Future studies should determine why old murine BMMs express higher CD38 compared to young controls after bacterial infection. Next, the oral pathogens (*Aa* or *Pg*) do not represent the entire periodontal pathogenic flora whose inflammatory response underlies periodontal disease. There are many other oral bacterial pathogens, including *Treponema denticola*, *Tannerella forsythia*, *Fusobacterium mecrophorum*, and *Prevotella intermedia* [49]. Future studies should determine if old murine BMMs could enhance CD38 levels and reduce NAD^+^ levels after infection with other oral pathogens. Additionally, aging patients have various age-associated diseases (including atherosclerosis, neurodegenerative diseases, autoimmune diseases, and type 2 diabetes). Future studies should determine if old murine BMMs displayed higher CD38 in response to other stimuli and if the inhibition of CD38 by 78c could reduce CD38, alleviate inflammation, and reduce oxidative stress induced by other stimuli. Furthermore, since we only conducted in vitro studies, future in vivo studies need to determine if treatment with 78c could alleviate periodontal inflammation, attenuate alveolar bone loss, and reduce oxidative stress in old animals with periodontitis.

Previously, CD38 monoclonal antibodies (including daratumumab and isatuximab) have been used to treat patients with multiple myeloma [50]. Although CD38 monoclonal antibodies inhibited the proliferation and survival of tumor cells, they also caused substantial side effects on anti-tumor NK cells [51]. We also observed an anti-proliferative effect in high-dose (10 μM) 78c-treated murine BMMs compared to vehicle-treated controls. Since CD38 is highly expressed in immune cells, the inhibition of CD38 could suppress immune responses by inhibiting the proliferation and survival of these immune cells.

## 4. Materials and Methods

### 4.1. Animals and Reagents

Thirty old (18-month-old) and thirty young (2-month-old) male C57BL/6J mice were purchased from Jackson Laboratory (Bar Harbor, ME, USA). Young female and male mice were bred to generate 2- to 3-month-old young control mice. The criteria for experimental animals were that the young mice were healthy and below 12 weeks old and the old mice were healthy and above 18 months old. All animal procedures were approved by the Institutional Animal Care and Use Committee (IACUC) at the Medical University of South Carolina and were carried out in strict accordance with the guidelines of the National Institutes of Health (NIH) for the care and use of laboratory animals. The CD38 inhibitor (78c) was purchased from Tocris Bioscience (Minneapolis, MN, USA) and dissolved in dimethyl sulfoxide (DMSO) as previously described [32]. An equal volume of DMSO (as compared to 10 mM 78c) was diluted in cell culture media and served as a vehicle control.

### 4.2. Generation of Bone Marrow-Derived Monocytes and Macrophages (BMMs)

Murine bone marrow cells were harvested from old (18-month old) or young (2- to 3-month-old) male C57BL/6J mice by flushing bone marrow cells from the tibia and femur using 10 mL cell culture media [complete minimal essential media (MEM)-α supplemented with 10% FBS, 100 U/mL penicillin and 100 µg/mL streptomycin] (Thermo Fisher Scientific, Waltham, MA, USA) as previously described [32]. To remove tissue debris, the flushed murine bone marrow cells were filtered through a 40 μM nylon cell strainer (Thermo Fisher Scientific). Then, murine bone marrow cells were cultured in a complete MEM-α media supplement with 20% L929 conditioned media (containing macrophage colony-stimulating factor, M-CSF) [32] for three days. The attached bone marrow stromal cells were discarded. The suspended cells were transferred to new cell culture plates and cultured in complete MEM-α media supplement with 20% L929 conditioned media for another seven days until cells were differentiated into attached BMMs.

### 4.3. Treatment with shRNA Lentivirus Vectors

The CD38 shRNA and control shRNA were generated as previously described [32]. Briefly, human embryonic kidney cells (HEK 293 cells) were transfected with a CD38 shRNA plasmid DNA (TRCN0000006832, Milipore Sigma, Burlington, MA, USA) or a control shRNA plasmid DNA (SHC002, Millipore Sigma) DNA along with lentiviral packaging plasmids pCMV-VSV-G (Addgene, Cambridge, MA, USA) and pCMV-dR 8.2dvpr (Addgene) using a lipofectamine 2000 transfection reagent (Thermo Fisher Scientific). Three days after DNA transfection, the supernatant was collected. Lentiviral vectors were obtained by ultracentrifation at 25,000 rpm for 1.5 h at 4 °C using a Beckman ultracentrifue (Beckman Coulter, Indianapolis, IN, USA) and followed by resuspension of viral pellets in serum-free DMEM media. Before lentiviral infection, murine BMMs were lifted by treating with 10 mM EDTA and plated in new cell culture dishes. Murine BMMs were infected with lentiviral vectors (MOI 10) for 3 days.

### 4.4. Bacterial Culture and Cellular Infection

The oral bacterial pathogens *Aggregatibacter actinomycetemcomitans* (*Aa*, ATCC 43718) and *Porphyromonas gingivalis* (*Pg*, ATCC 33277) were purchased from the American Type Culture Collection. Bacterial culture was described previously [32,52]. Briefly, *Aa* was cultured in brain–heart infusion broth (Fisher Scientific, Suwanee, GA, USA) at 37 °C with 10% CO_2_. *Pg* was cultured in tryptic soy broth (Becton Dickinson, Sparks, MD, USA) supplemented with yeast extract (Becton Dickinson, 1 mg/mL), menadione (Chem-Implex Int’l Inc., Wood Dale, IL, USA, 1 μg/mL), and hemin (Millipore Sigma, St. Louis, MO, USA, 5 μg/mL) at 37 °C under anaerobic conditions. Before infection, the bacterial pellets of *Pg* or *Aa* were washed and resuspended with PBS. One day before infection, the cell culture media were changed with MEM-α media with 5% FBS and 20% L929 conditioned media without antibiotics. Murine BMMs were infected with *Aa* or *Pg* with multiplicity of infection (MOI) of 20 to induce detectable cytokine expression in the BMMs. A control group of cells were not infected with bacteria.

### 4.5. NAD^+^ Assay

The NAD^+^ levels were determined as previously described [32] using a NAD^+^/NADH cell-based assay kit according to the manufacturer’s instructions (Cayman Chemical, Ann Arbor, MI, USA). The NAD^+^ levels were calibrated by cell growth and viability determined using a CellTiter 96 Aqueous One Solution Cell Proliferation Assay (Promega, Madison, WI, USA).

### 4.6. Enzyme-Linked Immunosorbent Assay (ELISA)

IL-1β, IL-6, and TNF-α protein levels were determined using ELISA kits (R&D Systems, Minneapolis, MN, USA) as previously described [32]. The concentration of cytokines was normalized by protein concentration in cell lysate.

### 4.7. RNA Extraction and Real-Time PCR

RNA was isolated from murine BMMs as previously described [32]. Briefly, cells were lysed by TRIZOL^®^ (Thermo Fisher Scientific). Complementary DNA was synthesized using a TaqMan reverse transcription kit (Life Technologies, Carlsbad, CA, USA) using total RNA (1 μg). Real-time PCR was performed as previously described [32] using the following conditions: 50 °C for 2 min, 95 °C for 10 min, and 40 cycles of 95 °C for 15 s, and 60 °C for 1 min. The following amplicon primers were purchased from Life Technologies: CD38 (Mm00483143_m1), Nox1 (Mm00549170_m1), Sod1 (Mm01344233_g1), Gpx4 (Mm04411498_m1), Prdx1 (Mm01621996_s1), Txnrd1 (Mm00443675_m1), Cat (Mm00437992_m1), Sirt1 (Mm01168521_m1), Parp1 (Mm01321084_m1), and β-actin (Mm02619580_g1). Amplicon concentration was determined using threshold cycle values compared with standard curves for each primer. Sample mRNA levels were normalized to an endogenous control β-actin expression and were expressed as fold changes compared with the control groups.

### 4.8. Western Blot Analysis

Western blot were performed as previously described [32]. Briefly, proteins were extracted from cells using a RIPA^®^ cell lysis buffer (Cell Signaling Technology, Danvers, MA, USA). Total protein (25 μg) was loaded on 10% Tris-HCl gels, electro-transferred to nitrocellulose membranes. The nitrocellulose membranes were blocked 1 h with 5% milk, washed, and incubated overnight at 4 °C with primary antibodies (1:500 to 1:1000). The antibodies to CD38, p-PI3K, p-ERK, p-JNK, p-p38, p-NF-κB p65, Sod1, Gpx4, Trdx1, Txnrd1(Trxr1), Cat, Sirt1, Parp, and pan-actin were purchased from Cell Signaling Technology. An antibody to Nox1 was obtained from Invitrogen (Carlsbad, CA, USA). After washing, the nitrocellulose membranes were incubated for 1 h at room temperature with horseradish peroxidase-conjugated secondary antibodies (Cell Signaling Technology). Finally, the membranes were incubated with SuperSignal West Pico Chemiluminescent Substrate (Thermo Fisher Scientific) for 5 min at room temperature. Digital images and protein densitometry were analyzed using a G-BOX chemiluminescence imaging system (Syngene, Frederick, MD, USA).

### 4.9. Statistical Analysis

Data were checked for normality using a QQ plot. The data were analyzed using a one-way ANOVA with Dunnett’s or Tukey’s multiple comparisons tests. All statistical tests were performed using GraphPad Prism software (Version 10.4.0, GraphPad Software Inc., La Jolla, CA, USA). Values were expressed as means ± standard error of the means (SEM) of multiple (3 to 5) independent experiments. A *p*-value of 0.05 or less was considered significant.

## 5. Conclusions

In this study, we observed abnormal high CD38 protein levels and low NAD^+^ levels in old murine BMMs after infection with oral pathogens compared to young controls. The abnormal high protein levels of CD38 were not directly correlated with the activation of NF-κB, PI3K, and MAPK protein kinases nor the amount of pro-inflammatory cytokines (IL-1β, IL-6, and TNF-α) released in old murine BMMs. The inhibition of CD38 by a CD38 inhibitor (78c) in old murine BMMs reduced CD38 expression, enhanced NAD^+^ levels, and subsequently alleviated oxidative stress and enhanced longevity genes (Sirt1 and Parp) induced by oral pathogens. Treatment with 78c also suppressed the activation of NF-κB, PI3K, and MAPK protein kinases induced by oral pathogens and alleviated pro-inflammatory cytokine (IL-1β, IL-6, and TNF-α) expressions. Additionally, treatment with 78c suppressed osteoclastogenesis and bone resorption induced by RANKL [32]. These results support that the inhibition of CD38 by 78c is a promising therapeutic approach to treat older patients with periodontitis to inhibit periodontal inflammation, attenuate alveolar bone resorption, alleviate oxidative stress, and prolong the health span of human beings.

## Data Availability

The data presented in this study are available upon request from the corresponding author.
